# The Jejunal Microbiota in Patients With Chronic Pancreatitis: Results From a Pilot Study

**DOI:** 10.1016/j.gastha.2026.100907

**Published:** 2026-03-03

**Authors:** Priyanka Sarkar, Subhaleena Sarkar, Misbah Unnisa, Anirudh Pratap Singh, Pradev Inavolu, Hardik Rughwani, Aparna Jakkampudi, Shashidhar Jaggaiahgari, D. Nageshwar Reddy, Rupjyoti Talukdar

**Affiliations:** 1 Pancreas Research Group and Division of Gut Microbiome Research, Wellcome DBT India Alliance Laboratories, Institute of Translational Research, Asian Healthcare Foundation, Asian Institute of Gastroenterology, Hyderabad, Telangana, India; 2 Department of Medical Gastroenterology, Asian Institute of Gastroenterology (AIG Hospitals), Hyderabad, India

**Keywords:** Jejunal Microbiome, Dysbiosis, Diabetes, Chronic Pancreatitis, Tight Junctional Proteins, Plasma Metabolites, Short Chain Fatty Acids

## Abstract

**Background and Aims:**

Chronic pancreatitis (CP) is associated with several systemic metabolic abnormalities including diabetes. While the colonic microbiota and its association with diabetes in CP have been reported, the specific composition of the small intestinal microbiota and its function in CP remains poorly understood. In this pilot study, we primarily aimed to characterize the jejunal microbiota in patients with CP and explore potential associations with diabetes.

**Methods:**

Jejunal aspirates were collected in a RNAlater-containing sterile container from 29 patients with CP and 10 controls. The samples were then snap lysed followed by metagenomic DNA extraction. Next-generation sequencing was performed for the variable region 3–4 of the 16SrDNA in Illumina MiSeq. After quality control, microbial profiling and functional analysis were conducted using standard bioinformatics pipelines. We also evaluated tight junction integrity in jejunal biopsy samples using immunofluorescence. Furthermore, we assessed for plasma and stool metabolites.

**Results:**

Patients with CP exhibited higher abundances of *Prevotella vespertina*, *Prevotella oris*, and *Prevotella salivae*, while controls demonstrated higher abundances of *Prevotella scopos*, *Veillonella*, *Rothia*, and *Lachnospiraceae*. Immunofluorescence showed decreased expression of the tight junction protein occludin in the jejunal mucosa of CP diabetic (CPD) patients compared to endoscopic controls (EC) (p.corr. CPD-EC = 0.012). No differences were seen between CP nondiabetic and endoscopic controls, and between the CP subgroups (CPND-EC = 0.29 and CPD-CPND = 1 respectively). Overall, there were significant plasma metabolomic abnormalities in patients with CP and a trend toward reduction of butyrate in the stool samples of the CP patients with diabetes.

**Conclusion:**

Our observations suggest alterations in the jejunal microbiota and mucosal barrier function in CP. These were associated with lower fecal butyrate. This may contribute to the pathogenesis of associated metabolic complications in CP. Further large-scale longitudinal and mechanistic studies are needed to validate our findings.

## Introduction

Chronic pancreatitis (CP) is a progressive inflammatory condition characterized by recurrent abdominal pain, pancreatic exocrine insufficiency (PEI), and endocrine dysfunction. PEI results from the destruction of pancreatic acinar cells and manifests as maldigestion of fats and other nutrients. Endocrine abnormalities initiate as beta cell dysfunction that culminate in frank diabetes. The composite of these factors contributes to malnutrition and metabolic dysfunctions in patients with CP.

Ours and others’ previous studies have suggested a link between gut microbial dysbiosis and diabetes in CP.^1^, ^2^, ^3^ These studies utilized fecal samples, which predominantly represented colonic bacteria. However, the specific alterations in the small intestinal, particularly jejunal, microbiota and their impact on metabolic abnormalities remain relatively unexplored. Our jejunum harbor a distinct microbial community that is significant for pancreatic health.^4^ The jejunum is the primary site for nutrient absorption and is in close proximity to the pancreas, receiving pancreatic enzymes and bile acids that influence microbial composition and activity.^5^ Alterations in the jejunal microbiome could therefore directly impact pancreatic function and inflammation. In patients with CP, several factors may contribute to jejunal microbial dysbiosis. Exocrine pancreatic insufficiency, which leads to maldigestion and malabsorption, impacts the availability of nutrients for the gut microbiota.^6^ Furthermore, changes in bile acid secretion and composition, often observed in CP, can also affect the microbial community structure in the small intestine.^7^^,^^8^ These alterations may promote the overgrowth of pathogenic bacteria, increase intestinal permeability, and trigger local and systemic inflammation, potentially exacerbating pancreatic injury and the resulting metabolic derangements.

Evaluation of the jejunal microbiota in patients with CP is therefore crucial for understanding the complex interplay between the gut and the pancreas. Characterizing the specific microbial changes associated with CP, identifying potential pathogenic mechanisms, and exploring therapeutic interventions targeting the jejunal microbiota may offer novel strategies for managing this challenging condition.

To investigate this, we conducted the current pilot study to characterize changes in the jejunal microbiota in patients with CP and assess their association with metabolic abnormalities, namely diabetes mellitus. We also evaluated alterations in plasma metabolites and fecal short chain fatty acids (SCFAs) in the patients based on the diabetic status.

## Methods

### Patient Recruitment

**Figure 1:**
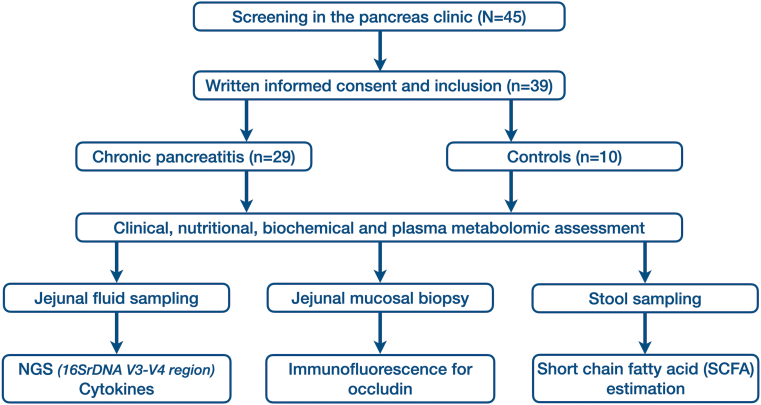
Flowchart highlighting the study flow.

**Supplementary Figure 1:**
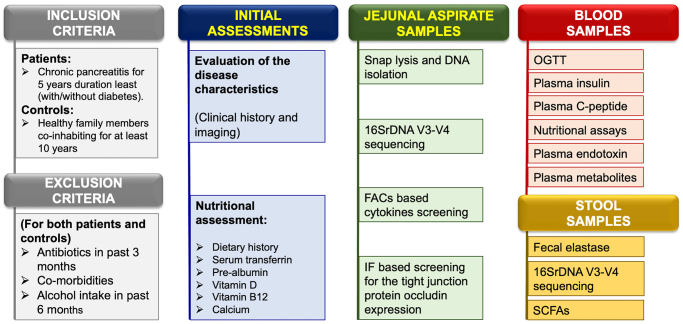
Flowchart highlighting the detailed study design.

Approval from the Institutional Review Board of the Asian Institute of Gastroenterology (IRB approval no. AIG/AHF IRB: 17/2017; dated April 27, 2017) was obtained prior to initiating the study. All participants provided written informed consent. All procedures adhered to standard guidelines. Figure 1 and Supplementary Figure 1 highlight the overall study flow and detailed overview of the study design, including inclusion and exclusion criteria.

We screened adult patients (aged between 18 and 50 years) of both genders with a diagnosis of CP for at least 5 years with diabetes (CPD) or without diabetes (CPND) at the Pancreas Clinic of the Asian Institute of Gastroenterology, Hyderabad, India (between 2022 and 2023) for inclusion into the study. We considered healthy family members cohabitating with the patients for at least 10 years to serve as endoscopic controls (EC).

We excluded patients who used antibiotics within the past 3 months from study enrollment, had comorbidities (irritable bowel syndrome, inflammatory bowel diseases, chronic liver disease, obesity, recent diarrhea), and consumed alcohol within the past 6 months from enrollment.

### Definitions and Study Methods

CP was diagnosed based on the Mayo Criteria. Comprehensive clinical history was recorded from both patients and controls. Morphological evaluation of CP was assessed using contrast-enhanced computed tomography and/or magnetic resonance cholangiopancreatography and/or endoscopic ultrasound. All screened healthy participants underwent a transabdominal ultrasound to evaluate for any pancreas and liver abnormalities.

Glucose intolerance and diabetes were defined according to the 2014 American Diabetes Association criteria.

Clinical assessment of nutrition was conducted with dietary history and subjective global assessment, which is a standardized and validated method to evaluate nutritional status. Biochemical assessments were conducted to evaluate the circulating levels of transferrin, vitamins B12 and D, and hemoglobin. Glucose metabolism was evaluated by oral glucose tolerance tests, C-peptide, plasma insulin, and hemoglobin A1c measurements. We also evaluated plasma endotoxin levels for both controls and cases. Patients with steatorrhea or fecal elastase value of < 200 μg/g were considered to have PEI. Fresh stool samples were collected to assess fecal elastase levels.

### Biochemical Assays

Oral glucose tolerance test was performed using a 75 g glucose load. Blood glucose levels were determined using the Glucose Oxidase-Peroxidase (GOD-POD) method with an ERBA Glucose kit (Transasia Bio-Medicals Ltd, HP, India). Hemoglobin A1c was measured by high performance liquid chromatography on an National Glycohemoglobin Standardization Program-certified automated analyzer (Bio-Rad). Plasma insulin and C-peptide concentrations were assessed using a Sandwich Electrochemical Immunoassay on a Roche Cobas e 601 Immunoassay Analyzer. Serum vitamin D, vitamin B12, and calcium levels were determined by electrochemical immunoassay and colorimetric methods, respectively, using Roche Cobas e 601 and UniCel DxC 800 analyzers. Serum transferrin and prealbumin were measured by immunoturbidimetry on a Random Imola analyzer. All assays adhered to manufacturer guidelines.

### Fecal Elastase Assay

Fecal elastase was assayed in fresh fecal samples using the solid phase double-sandwich ELISA based kit (Code no. G09038; Bioserve Diagnostics, Rostock, Germany) as per the manufacturer’s instructions.

### Jejunal Fluid Aspiration

A pediatric colonoscope (length 168 cm, instrument channel 3.2 mm) was gently advanced into the distal duodenum and proximal jejunum. Free fluid was gradually drained from all the mucosal folds. In the absence of free fluid, 10 ml of distilled water was instilled, followed by delayed aspiration after 3 to 5 min. The aspirate was collected in a separate sterile container connected to the suction port.

### Fluorescence-Activated Cell Sorting–Based Quantification of Inflammatory Cytokines

We evaluated cytokine concentrations in jejunal aspirate using Becton Dickinson (BD) cytometry bead array (CBA) human inflammatory cytokines kit (Cat No: 551,811, BD Biosciences) based on the manufacturers’ protocol. Briefly, 6 bead populations with distinct fluorescent properties were coated with antibodies specific for interleukin (IL)-8, IL-1b, IL-6, IL-10, tumour necrosis factor-a, and IL-12P70. Samples were mixed with these beads and then exposed to a PE-conjugated reagent. The resulting mixture was analyzed using a BD fluorescence-activated cell sorting (FACS) Lyrics flow cytometer and the data were processed with FCAP software.

### Immunofluorescence-Based Screening of the Tight Junctional Protein (Occludin)

Localization of the tight junction protein occludin in jejunal mucosa was determined by immunofluorescence microscopy.^9^ Briefly, intestinal tissue sections were deparaffinized with xylene and rehydrated using a graded ethanol concentrations, followed by rinsing twice with distilled water. Sections were then permeabilized using 90% chilled methanol for 30 mins. Antigen blocking was carried out using 5% bovine serum albumin in PBS for 30 min to prevent nonspecific binding. The sections were subsequently incubated overnight at 4 °C with primary antirabbit antibody (1:100, Abcam). After incubation with the primary antibody, the slides were thoroughly washed multiple times to remove unbound antibody, followed by 1 h incubation at room temperature in the dark with fluorescent tagged secondary antibody (1:350). After additional washes, the sections were mounted with a fluoroshield mounting medium containing DAPI (Abcam). Imaging was performed using the EVOS FL Auto Microscope (Thermo Fisher Scientific). Target protein expression was quantified using the IMAGE J software^10^ (NIH) by analyzing 5 randomly selected fields per section, and positive expression values were reported as mean with standard error of mean.

### Metagenomic DNA Isolation

For DNA extraction, jejunal aspirate and stool samples were collected in sterile containers, containing RNAlater (cat. no. 761016, Qiagen, Germany), and were immediately stored at −80 °C until further processing. Jejunal biopsies were collected in sterile containers containing 10% neutral buffered formalin (Merck) and kept at room temperature (25 °C) for further use. Genomic DNA was isolated from the jejunal aspirates as well as stool specimen (200 mg) using the QIAmp Fast DNA Stool Mini kit (Qiagen, cat. No. 51604) following the manufacturer’s instructions. We also took snap lysed fluid for the microbiome study since the control samples were exhibiting low DNA quantity on preliminary runs. DNA concentrations were determined using Nanodrop 2000 spectrophotometer (Thermo Scientific, IL), and the purity was assessed based on absorbance at A260/A280.

### Next-Generation Sequencing on Illumina MiSeq Platform

We considered variable region 3–4 based amplicon analysis for exploring the jejunal and fecal microflora. Illumina 16SrDNA gene libraries were constructed for each sample by 30 cycles of PCR amplification with 16S Illumina Amplicon primers at an annealing temperature of 55 °C. The cycle number was adjusted to account for the quantity of bacterial DNA in the sample.^11^

The Nextera DNA flex library kit was utilized in this study. The 5′ adaptor sequences each were tagged with a distinct bar-coded Illumina Nextera forward and reverse adaptor primer, and the Illumina Amplicon primer sequences we used carry the degenerative DNA sequences in the 3′-terminal that match extremely conserved sequences flanking the V3 and V4 hypervariable regions of all bacterial 16S rDNA genes.^11^ A distinct pair of bar-coded primers was used to identify each library.^11^

For each patient, Illumina MiSeq sequencing yielded roughly 250 bp of sequence from forward and reverse strands of each molecular cluster template to assemble a ∼460 bp 16S variable 3–4 region DNA sequence. Using bar-coded sequence tags, data were sorted back into individual patient samples and analyzed with amplicon metagenomic downstream analysis with the updated MiSeq Reporter (v2.5) software package.^12^

### Bioinformatics Analysis

**Supplementary Figure 2:**
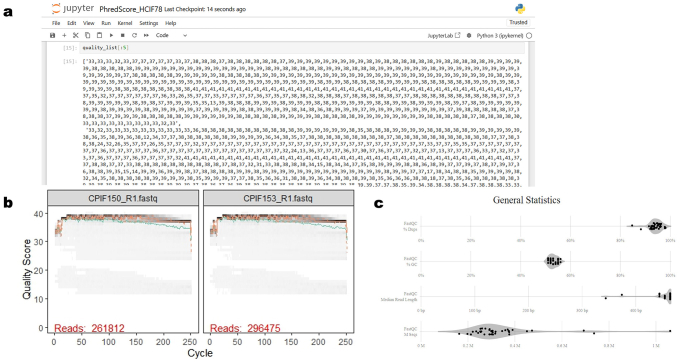
(A) Snapshot of *phred scores* depicting the data quality. (B) Data prequality and (C) postquality check and trimming the bad reads.

We used Quantitative Insights Into Microbial Ecology (ver. 2–2023.5)^13^ for further downstream analysis. We first checked the quality score by constructing a program using ‘for loop’ with the ‘while’ condition in python to understand the phred score distribution across the sequences observed sample wise (Supplementary Figure 2A). Parallelly, we cross-checked the sequence quality using Divisive Amplicon Denoising Algorithm 2 (ver. 1.31.0; Supplementary Figure 2B).^14^ We then used AdapterRemoval (ver. 2.3.2)^15^ to trim the adaptors and low-quality sequences (Supplementary Figure 2C). After trimming the reads, we stitched them followed by de-noising and inferring exact amplicon sequence variants using Divisive Amplicon Denoising Algorithm 2 (ver.1.26.0; Rcpp: 1.0.10, RcppParallel: 5.1.6) in R (ver. 4.2.3). After receiving the amplicon sequence variant files, we classified the feature taxonomic profiling using ‘sklearn’^16^ classifier with reference database Greenegenes (ver 2.0).^17^ For functional profiling analysis, we used Phylogenetic Investigation of the Communities by Reconstruction of Unobserved States (v.1) using Kyoto Encyclopedia of Genes and Genomes (KEGG) and KEGG Orthology database. We used species taxon abundances to evaluate the functional annotations.^18^^,^^19^

### Metabolome Analysis of the Plasma and Stool Samples

We conducted a pilot-targeted plasma metabolome analysis to understand the key alterations in specific metabolites, especially amino acids, and lipids, based on our previous studies.^20^ Briefly, we collected 5 ml of blood in a K3 EDTA container, centrifuged it for 10 min at 2000 × *g* at 4 °C and separated the plasma samples. The samples were then snap-frozen with liquid N_2_ and later used for metabolomics analysis using an AbsoluteIDQ p180 kit (Biocrates, Austria) following the manufacturer’s instructions.

For analyzing the fecal SCFAs, we followed the method described earlier.^21^ Briefly, we extracted 50 mg of the fecal samples using 200 μL of methanol (Lichrosolve, Merck, Darmstadt, Germany) for 15 h at room temperature. We then centrifuged the samples at 12,000 rpm at 4 °C for 5 min and collected the supernatants. Following this, we derivatized the samples with N-methyl-N-(trimethylsilyl) trifluoroacetamide. The prepared samples were then injected to a Shimadzu GC 2010 plus with triple quadruple MS (TP-8030) fitted with an EB-5MS column based on the protocol described earlier.^21^

### Statistical Analyses

Since, this was, to the best of our knowledge, the first study to understand the indigenous jejunal microbiome in CP, we did not perform any formal sample size calculation. A database was generated in Excel for further statistical analyses. We used the Statistical Package of Social Sciences (SPSS, IBM SPSS 20; SPSS Inc, Chicago, IL), Paleontological Statistics Software (version 3.11 for Mac) and R studio (R version 4.3.1 [2023–06–16 ucrt]) platforms for conducting the statistical analyses. Continuous clinical data were expressed as mean (±standard deviation), while categorical data were expressed as proportions.

Bacterial alpha diversity was represented as Chao1 (species richness) and Shannon (species evenness) indices.^22^^,^^23^ Beta diversity was computed using Bray-Curtis distance matrix^24^ at species taxon level and represented using principle component analysis (PCS) and principle coordinate analysis plots as applicable. We used Quantitative Insights Into Microbial Ecology 2 (ver. 2023.5)^13^ for computing the bacterial diversity. We used R (FactoMineR package)^25^ for PCA plot generation and MicrobiomeAnalyst^26^ to construct the Rarefaction curves of each sample. Significance testing for the between-group clustering of the samples in PCA was checked using 1-way permutational multivariate analysis of variance (PERMANOVA).^27^ Additionally, we performed agglomerative hierarchical cluster analysis with bootstrapping to 1000 to understand the distance/differences in microbial profile between each samples. Differences in the microbiome profiles were tested using the nonparametric Kruskal–Wallis test, with Bonferroni corrections in SPSS (IBM). The bacterial abundances were expressed as box and whisker plots based on their relative abundances of different taxa and heatmaps. Heatmaps were constructed based on the z-score, which was calculated according to the relative abundances of the microbial taxa. Microbial dysbiosis index was calculated as the log[total abundance of organisms increased in the group X/total abundance of the organisms decreased in the group X] as per the formula described earlier.^16^ For PCA plot generation to evaluate metabolite, we used MetaboAnalyst.^28^ For evaluating the differences in the metabolomics profiles, we used PERMANOVA, and for evaluating the specific altered metabolites, FACS, and IF data, we used Mann–Whitney *U* and Kruskal–Wallis tests, with Bonferroni correction as applicable. We considered the adjusted ‘p’ value (denoted as p [corr.]) of < 0.05 as statistically significant.

## Results

### Patient Characteristics

As shown in the Figure 1, we initially screened 45 participants, out of which we recruited 29 patients and 10 controls who fulfilled enrollment criteria. Patient details are presented in Table 1. Among the 29 patients with CP, 15 diabetes. We did not observe any significant differences in the duration of CP between patients with and without diabetes. Diabetic CP patients had lower plasma C-peptide concentrations as compared to the nondiabetic group, though not statistically significant [mean (±standard deviation): CPD-1.28 (0.77) and CPND-1.9 (1.91) ng/ml, *P* = .9]. The mean age of onset of diabetes was 34.4 (±10.01) yrs. There was no statistically significant difference in the nutrition status between CP diabetics compared to CP nondiabetics (*P* = 1.0). We did not find any significant differences in transferrin and vitamin D concentrations between CPD and CPND.

**Table 1:** Characteristics of the Participants Enrolled in the Study

Parameters	Control (n = 10)	Chronic pancreatitis without diabetes (n = 14)	Chronic pancreatitis with diabetes (n = 15)
Demographic characteritics			
Age (yrs), mean (SD)	38.3 (7.3)	33.9 (13.2)	35.5 (10.4)
Male gender, n (%)	8 (80)	9 (64.3)	11 (73.3)
Nonvegetarian diet, n (%)	10 (100)	10 (71.4)	13 (86.7)
Disease characteristics			
Duration of CP diagnosis at enrollment (yrs) (mean; SD)	Not applicable	5.42 (3.6)	8.35 (6.8)
Etiology (alcohol/idiopathic), n (%)		5 (35.7)/10 (71.4)	7 (46.7)/7 (46.7)
Gross pancreatic atrophy, n (%)		7 (50)	8 (53.3)
Pancreatic calcification/calculi, n (%)		10 (71.4)	8 (53.3)
Pancreatic pseudocyst, n (%)		0	0
Endoscopic/surgical intervention, n (%)		11 (78.6)	9 (60)
Fecal elastase positivity [n (%)]		8 (57.1)	10 (66.7)
Nutritional characteristics			
Subjective global assessment (A/B/C) [n]	Not done	A-13, B-1, C-1	A-11, B-3, C-0
BMI at presentation (mean; SD)		21.95 (2.9)	22.36 (4.59)
Transferrin (mg/dL) (mean; SD)		285.75 (53.17)	285.92 (75.80)
Vitamin D (U/L) (mean; SD)		15.97 (7.4)	17.47 (11.69)
Vitamin B12 (pg/mL) (mean; SD)		465.16 (270.3)	350.35 (172.42)
Glycemic status			
Duration of diabetes (yrs) (mean; SD)	Not done	NA	3.93 (2.91)
On insulin at the time of enrolment, n (%)		NA	8 (53.3)
Fasting blood glucose (mg/dL) (mean; SD)		99.5 (19.1)	184.73 (92.02)
Postprandial blood glucose (mg/dL) (mean; SD)		163.75 (66.4)	350.90 (167.97)
Fasting insulin (U/mL)(mean; SD)		10.58 (17.3)	6.18 (4.19)
Glycosylated hemoglobin (mean %; SD)		6.19 (1.0)	9.52 (3.22)

SD, standard deviation.

Intergroup differences between any of the variables are not statistically significant.

### Metagenomic DNA-Sequence Characteristics and Bacterial Diversity

**Figure 2:**
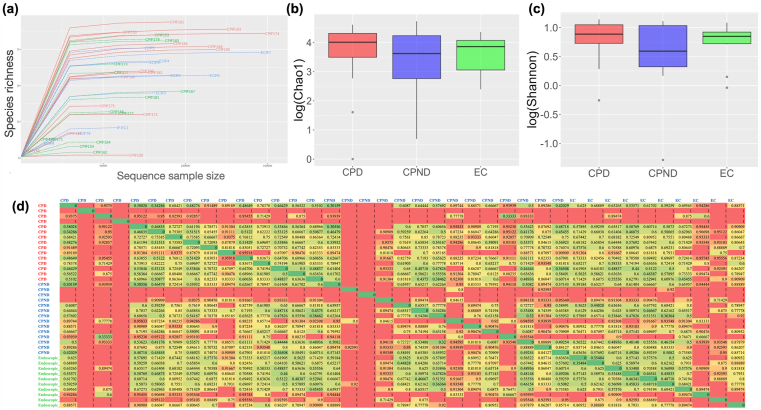
Bacterial richness and diversity with the progression of CP. (A) Rarefaction curves depicting the species richness of each sample. (B) Heatmap depicting the lower beta diversity (among the groups); box and whisker plot indicating (C) bacterial richness (Chao 1, Kruskal–Wallis test, [p(corr.) = 0.188]) and (D) Shannon’s alpha diversity in patients and controls (Kruskal–Wallis test, [p(corr.) = 0.793]. CPD, chronic pancreatitis with diabetes; CPND, chronic pancreatitis without diabetes; EC, endoscopic controls.

The metagenomic sequence characteristics are tabulated in Supplementary Table 1. Figure 2A shows the individual rarefaction curves of the participants. We did not observe any significant differences in species diversity in terms of the Chao1 index between the diabetic CP, nondiabetic CP, and control individuals (Figure 2B). Additionally, Shannon index (Figure 2C) and beta diversity (Whittaker’s index, Figure 2D) were also similar between the groups. Individual DNA sequence characteristics and diversity indices for the patients are presented in Supplementary Table 1.

### DNA-Based Microbiome Analysis of the Studied Samples

**Supplementary Figure 3:**
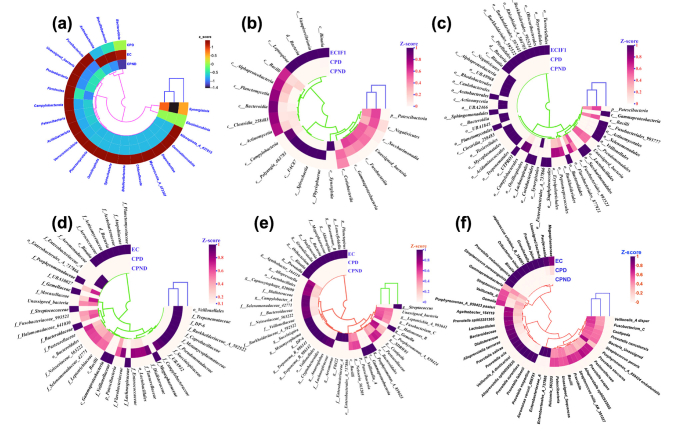
Bacterial abundance (phylum-species; A-F) in the metagenomic DNA isolated from the jejunal aspirate. CPD, chronic pancreatitis with diabetes; CPND, chronic pancreatitis without diabetes; EC, controls.

We opted for metagenomic DNA-based microbiome analysis for a few samples (CPD-6, CPND-5 and EC-1). As depicted in the Supplementary Figure 3, we observed that CPD group had different jejunal microbial profile compared to CPND and controls. Similar to the aspirate/snap lysed fluid, CPD group had a significantly higher abundance of unassigned bacteria as compared to CPND and controls. The control group had a higher abundance of Bacteroidota and Desulfobacterota. CPND group exhibited lower bacterial abundance as compared to the CPD group. We also observed lower metagenomic DNA and bacterial quantity in the EC group. However, we did not find any difference in alterations in bacterial species between CPD and CPND groups.

### Differences in Bacterial Taxa in Jejunal Fluid in the Study Groups

**Figure 3:**
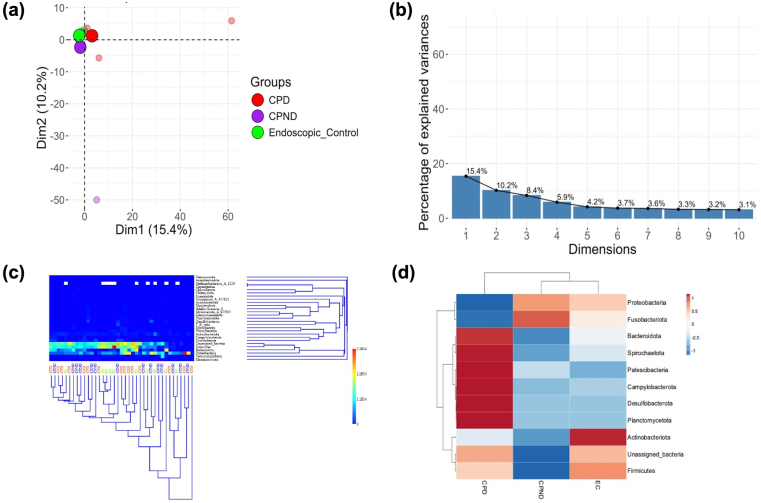
Clustering of gut microbiome at species level among the study groups. (A) Principal component analysis (PCA) of gut microbial species abundance showing distinct clustering in the 3 study groups. The prominent circle depicts centroid point of the bacterial profile of each group. (B) Scree plot for PCA analysis. (C) Heatmap with dendrogram showing hierarchical cluster analysis based on Euclidean similarity matrix (at phyla level, with bootstrapping to 1000) to measure closeness between individual samples based on their gut bacterial profile. (C) Phylum abundance in the three study groups. CPD, chronic pancreatitis with diabetes; CPND, chronic pancreatitis without diabetes; EC, endoscopic controls.

Figure 3A depicts the PCA plots showing a distinct clustering of the controls, diabetic CP, and nondiabetic CP at the species level taxa (PERMANOVA, *P* = .003). Figure 3B depicts the scree plot for the PCA analysis, while Figure 3C depicts the hierarchical clustering of individual samples within the 3 groups (at the phyla level).

We observed a total of 25 bacterial phyla, of which Firmicutes (27.11%), Proteobacteria (21.91%), Bacteroidetes (12.82%), Actinobacteriota (2.29%), Fusobacteriota (1.53%), and unassigned bacteria (30.9%) were predominantly observed in samples across the groups. As shown in Figure 3D, we observed an increased abundance of the phyla of Bacteroidetes from controls to nondiabetic CP. We observed a trend toward an increase in unclassified or novel bacteria in jejunal fluid in the diabetic CP group.

**Figure 4:**
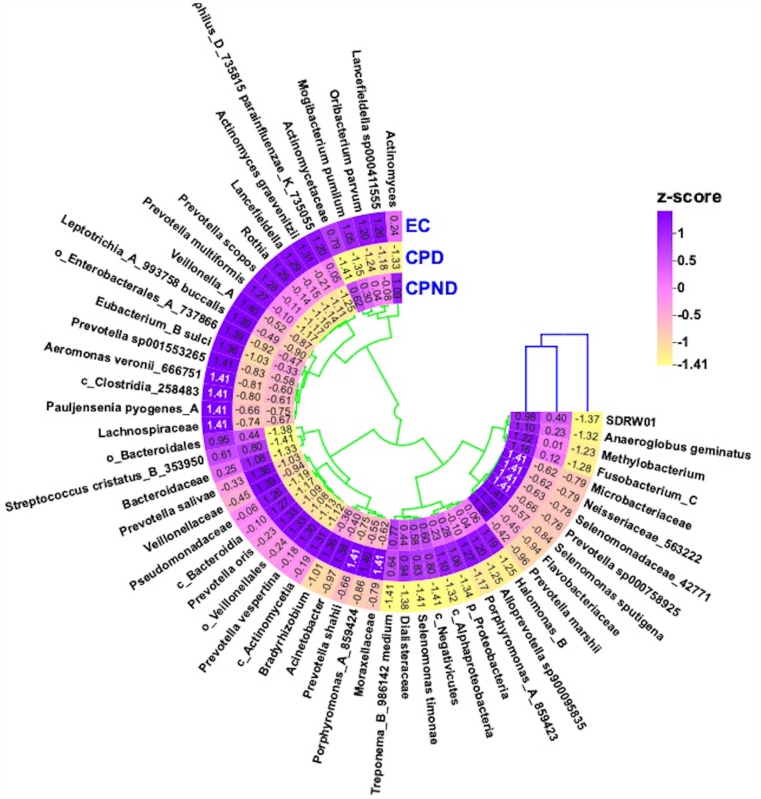
Heatmap depicting the altered bacterial species observed in the groups (z-score has been calculated based on the median values of the bacterial abundances). CPD, chronic pancreatitis with diabetes; CPND, chronic pancreatitis without diabetes; EC, endoscopic controls.

As shown in Figure 4, *Prevotella vespertina*, *Prevotella oris*, unassigned bacterial species under Pseudomonadaceae, *Prevotella salivae*, *Alloprevotella* sp. (strain-900095835), and Moraxellaceae had greater abundance in the CPD group. CPND group, on the other hand, was found to have a higher abundance of Fusobacterium_C, Neisseriaceae (strain-563222), *Methylobacterium*, Selenomonadaceae (strain-42771), and *Selenomonas sputigena*. The control group however had higher abundance of *Prevotella scopos*, *Veillonella*_A, Enterobacterales_A_(strain-737866), *Lancefieldella*, *Aeromonas veronii* (strain-666751), *Rothia*, *Leptotrichia* *buccalis* (strain-993758), and Lachnospiraceae. Even though we observed a trend toward species-level alteration between the cohorts, we did not observe any statistical significance. We then employed EdgeR-based analysis and observed a differential abundance of 58 microbial species between CPD vs CPND groups. Compared to the control group, 43 microbial species showed differential abundances in the CPD group, while 40 species were found to be differentially abundant in the CPND group (p-corr.<0.05, Supplementary Table 2).

Other than the diabetic status, we did not observe any associations of the dysbiosis with any of the demographic and other clinical or morphologic parameters.

### Differences in the Dysbiosis Index Between the Study Groups

**Supplementary Figure 4:**
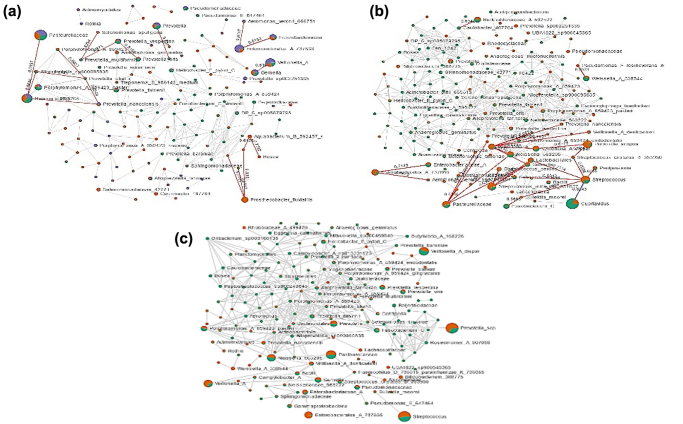
Microbial dysbiosis index: Network plot showing the most predominant bacteria correlating with each other in the group. Pie diagram depicting the median values of the bacteria observed in the groups. Each node represents a taxon with group specific color. The size of the node is based on the number of connected taxa observed with the specific microbial species. The group names have been highlighted in the image.

Supplementary Figure 4 depicts the Dysbiosis Index, which quantifies the extent of dysbiosis. The CPD group showed a deviation in terms of microbial dysbiosis index of 0.11 from controls, while CP nondiabetic showed a value of 2.01. Noticeably, when we calculated the Dysbiosis Index between CPD and CPND groups, it was found to be 4.42, indicating intraphenotypic differences in the microbiome profile of the cohorts.

### Functional Differences (In Silico) Among the Study Groups

**Figure 5:**
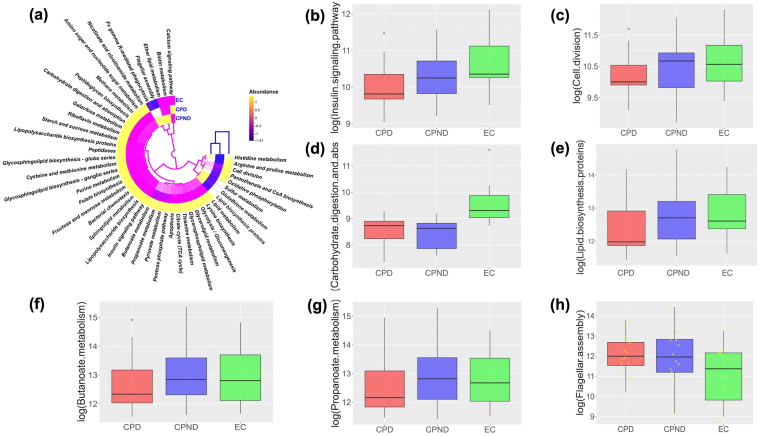
Functional differences (in silico) among the study groups. (A) Pathways which were found to be differentially expressed in the samples across the groups. (B) Insulin signaling pathway, (C) carbohydrate digestion, (D) lipid biosynthesis, (E) butanoate metabolism, (F) propanoate metabolism, (G) cell division, (H) flagellar assembly pathways. CPD, chronic pancreatitis with diabetes; CPND, chronic pancreatitis without diabetes; EC, endoscopic controls.

We then studied the functional pathways using the KEGG Orthology in the 3 groups and their corresponding bacterial abundance. The abundances of the pathways observed in the study are presented in Figure 5A. We observed a trend toward decreased insulin signaling pathways, carbohydrate digestion, and lipid biosynthesis pathways from controls to CPND to CPD patients (Figure 5B–E; Supplementary Table 3). Additionally, the pathways related to butanoate and propanoate metabolism were also lower in the CPD group (Figure 5F and G). Furthermore, cell division pathways were found to have lower while flagellar assembly pathways higher abundance in the CPD group followed by the CPND compared to the controls (Figure 5C and H).

### FACs-Based Analysis of the Cytokines in the Fluid Samples

**Supplementary Figure 5:**
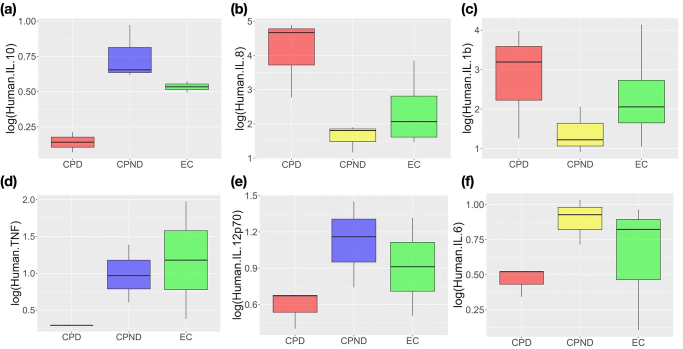
(A-F) Boxplots showing the concentrations of fluid cytokines—(A) IL-8, (B) IL-6, (C) IL-1b, (D) IL-10, (E) TNF-a, (F) IL-12—observed in the studied participants. CPD, chronic pancreatitis with diabetes; CPND, chronic pancreatitis without diabetes; EC, controls.

As shown in Supplementary Figure 5A–F, we observed a distinct trend in the elevation in plasma IL-8 (p.corr = 0.080) and IL-10 (p.corr = 0.051) concentration in CPD patients compared to CPND patients. IL-6 (p.corr = 0.218) and IL-12 (p.corr = 0.145) were similar in both the CP groups. Differences in the cytokine concentrations were not statistically different between the controls and the two CP groups.

### Immunofluorescence-Based Screening of the Tight Junctional Proteins (Occludin)

**Figure 6:**
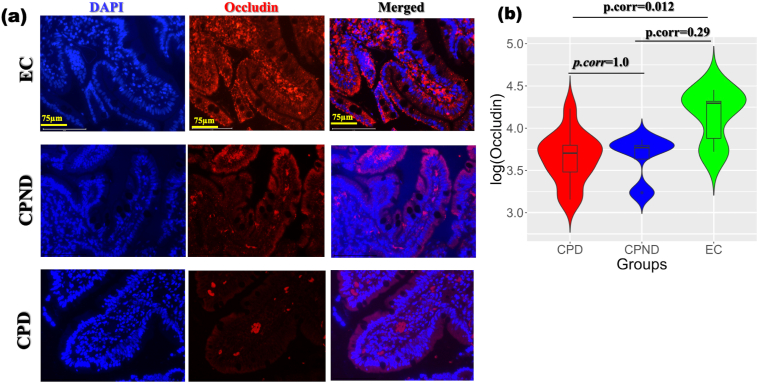
(A) Representative immunofluorescence (IF) images with a scale bar of 75 μm indicating occludin expression in the jejunal biopsies. Red fluorescence in the second column represents occludin expression, while yellow fluorescence in the last column are merged images of DAPI and occludin expressed in the tissue. (B) Violin plot showing the quantitative representation of occludin positivity, which was significantly higher in control, compared to the disease group (adj. p, EC/HC vs CPD = 0.012; EC vs CPND = 0.29, and CPD vs CPND = 1.0, respectively). CPD, chronic pancreatitis with diabetes; CPND, chronic pancreatitis without diabetes; EC, endoscopic controls.

Our analysis based on the intestinal histopathology revealed greater injury and inflammation of the jejunal segments in the disease groups. As shown in the Figure 6, the occludin expression was reduced in disease groups, which reached statistical significance between the CPD group and controls (p.corr = 0.012).

### Comparison of the Microbiome Patterns Across the Two Ecosystems of the Gastrointestinal Tract

**Supplementary Figure 6:**
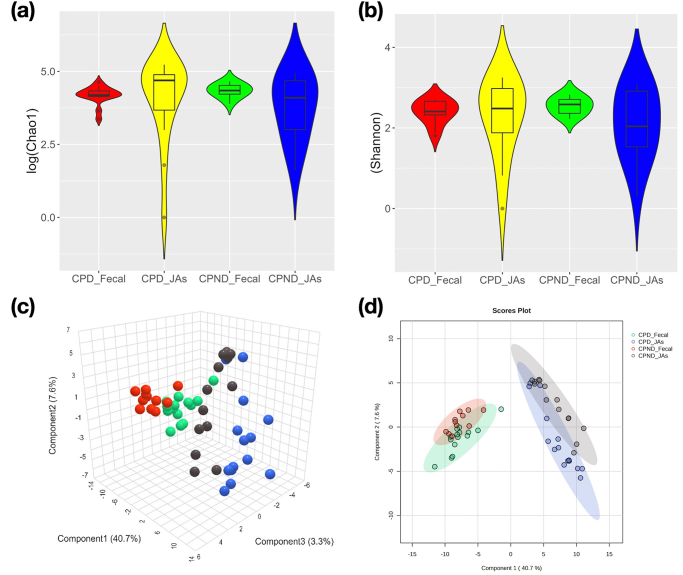
Microbiome profiles of jejunal and fecal sampling sites of the patients. (A) Partial least squares discriminant analysis (PLSDA) plot based on the abundance of the microbiome detected in the 2 sampling sites of the study participants. (B and C) Score plot and clustering patterns in different components to depict the distinct differences in the microbial composition across the sampling sites. CPD, chronic pancreatitis with diabetes; CPND, chronic pancreatitis without diabetes.

After evaluating the jejunal microbial characteristics, we compared jejunal microbiota profile with that of the fecal microbiota within the same patients. As shown in the Supplementary Figure 6, the microbiome load of the 2 ecosystems exhibited a distinct dissimilarity. The jejunal microbiota exhibited a higher alpha diversity than the fecal microbiota in the CPD group. However, in the CPND group, it was lower than the fecal niche (Supplementary Figure 6A and B). Principal coordinate analysis of the samples based on Bray-Curtis distances derived from the abundances of the microbial species highlighted a distinct segregation of jejunal and the fecal microbiota (Supplementary Figure 6C–E), which were further confirmed by presence of distinct phyla. We observed a total of 21 phyla in the jejunal samples, whereas 15 different microbial phyla were noticed in the fecal samples (Supplementary Table 1). In this study, we have only compared the microbial phyla between the 2 niches, considering the differences in the physiology, pH, availability of aerobic environment, nutrient availability, and other biochemical factors of the 2 sampling sites.

### Metabolomic Alterations in the Plasma and Stool Between Study Groups

**Figure 7:**
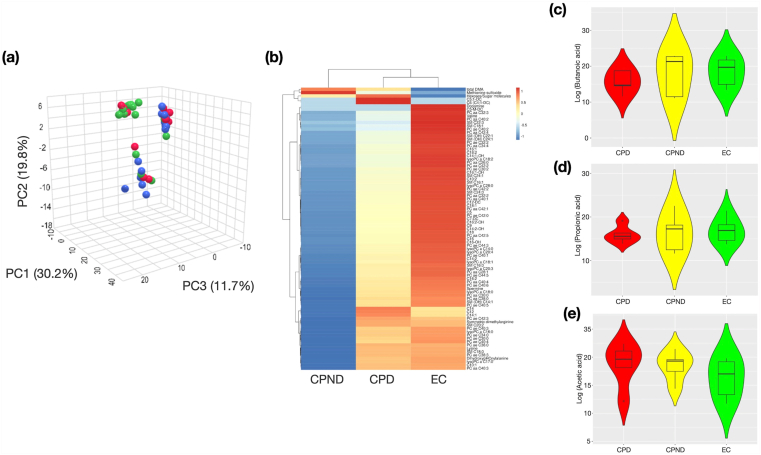
Plasma and fecal metabolomics profiles of the patients and controls. (A) PCA plot depicting the unique plasma metabolomics signature of the groups (PERMANOVA). (B) Heatmaps with dendrograms depicting the significantly altered plasma metabolites in the groups. The color scale represents the z-score, which has been calculated based on the median values of the groups. (C-E) Box and whisker plots represent the fecal metabolite profiles of (C) butanoic acid, (D) propionic acid, and (E) acetic acid concentrations (in ppm) observed in the groups. CPD, chronic pancreatitis with diabetes; CPND, chronic pancreatitis without diabetes; EC, endoscopic controls; PCA, principal component analysis.

**Supplementary Figure 7:**
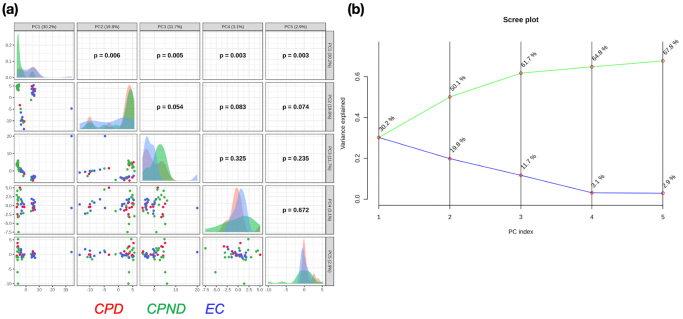
Plasma metabolites profiles of the patients. (A) PCA pair plots visually depicting the relationships and distributions among the principal components and (B) Scree plot depicting the optimal number of components or factors to retain in analyses.

Plasma metabolites and fecal SCFAs of individual participants are presented in Supplementary Table 4. We observed a total of 188 plasma metabolites which included amino acids, biogenic amines, lipids, and sugars molecules. As depicted in the Figure 7A, we observed different metabolome profiles between the study groups (PERMANOVA, p.corr CPD vs CPND = 0.070, CPD vs EC = 0.080 and CPND vs EC = 0.003). The PCA component pair plot and scree plot are shown in Supplementary Figure 7A and B. Further analysis revealed a total of 88 significantly altered metabolites, which are represented by heatmap (Figure 7B). On evaluating specific metabolites, we observed a higher abundance of monosaccharides in the CPD group (Figure 7B). A significantly reduced concentrations of valine, lysine, and dopamine were observed in the overall CP cohorts compared to the controls. Overall, lipids such as sphingomyelins and glycerolipids were more abundant in the control group, followed by CPD and CPND. Carnitine group of metabolites were also observed with a higher abundance in the control group. The CPD group, however, had a greater abundance of methionine sulfoxide and symmetric dimethylarginine in their plasma and CPND patients had higher abundance of total dimethylated arginine.

Furthermore, we evaluated fecal SCFAs and found a trend toward reduced concentrations of butyrate/butanoic acid and propionic acid concentrations in the CPD group compared to CPND and controls (Figure 7C and D). On the other hand, acetic acid was higher in the CPD group (Figure 7E).

## Discussion

In this study, we have shown that the jejunal microbiota is altered in patients with CP. The dysbiosis was most predominant in the patients who also had diabetes and was accompanied by alterations in the plasma metabolome and fecal SCFAs. Interestingly, the jejunal and fecal microbiota were distinct in the patients, thereby implying unique alteration in the jejunal microbiota which might independently contribute to the pathogenesis of the metabolic derangements in CP.

One of the key findings was the depletion of the health promoting organisms Lachnospiraceae, *Rothia*, and *Veillonella* in the patients with CP. Studies have found that gut Lachnospiraceae convert primary bile acids to secondary bile acids by 7α-dehydroxylation which in turn inhibit intestinal enteric pathogens.^29^^,^^30^ The species also have been reported to regulate mucosal immunity.^31^ *Rothia*, another gut commensal, is known to secrete diverse metabolites that interact with other commensals and the host digestive tract, to enhance intestinal biodiversity, improve glucose tolerance, and help recover colocyte vitality.^32^ Experimental studies have also proposed that supplementation of *Rothia* can repair injury in the intestinal mucosal barrier, optimize the intestinal permeability, inhibit the release of pro-inflammatory cytokines, and improve gut dysbiosis.^33^ The depletion of the respective bacterial species could be associated with impairment of the gut mucosal barrier integrity in our patients with CP. Additionally, the other predominant bacteria, *Veillonella*, can promote homeostasis by producing beneficial metabolites, specifically short-chain fatty acids (SCFAs), by lactate fermentation in human gut.^34^ We observed reduction of the beneficial SCFA butyrate in our patients with CP, especially those with diabetes. Therefore, reduction of *Veilonella* could have contributed to the disruption of gut mucosal barrier and the intestinal epithelial-bacterial interaction in our patients in this study.

We further observed that the patients with CP and diabetes had higher abundances of pathobiomes such as *P oris*, which has been reported to have the ability to cause infections in the other organs (eg, lung).^35^

The patients with CP but no diabetes displayed a higher abundance of *Fusobacterium*. Seminal studies have reported that several *Fusobacterium* species are associated with pancreatic cancer prognosis and CP severity.^36^ *S sputigena*, another abundant bacterium in the group, have been reported to have a positive association with chronic inflammatory diseases.^37^

Another key finding in our study is the downregulation of pathways related to insulin signaling, carbohydrate metabolism and lipid biosynthesis, all of which were more predominant among the CP patients with diabetes compared to those without and controls. These findings suggest a potential role of the dysbiotic jejunal microbiota in modulating metabolic pathways and contributing to the development of diabetes in CP.

Additionally, our immunofluorescence analysis of tight junctional proteins revealed a trend toward decreased occludin expression in patients with CP compared to controls. This can be associated with the lower abundance of butyrate in the stool samples of these patients, and suggests potential contribution to the alterations in intestinal permeability. Finally, the comparison of jejunal and fecal microbiomes in the same patients revealed distinct differences in microbial composition, highlighting the importance of site-specific analysis in understanding the role of the gut microbiota in CP.

Finally, we evaluated the plasma metabolomic profiles of the hosts to understand the impact of the microbial alteration at functional level. We observed a trend toward more sugars in CP patients with diabetes, which matches the endocrine characteristics. Essential amino acids such as lysine and valine was significantly lower in the patients with CP. L-lysine supplementation has been linked to improve glycemic control in both rat model and human.^38^^,^^39^ L-valine has also been reported to be a powerful stimulator of GLP-1 secretion in rodents model.^40^ These findings suggest a putative role of these amino acids in altering the insulin dynamics in our patients. In some in vivo investigations, dopamine, which was low in our CP patients, had been reported to either boost or reduce insulin secretion or resistance. However, human studies have shown that dopamine infusion had no negative effects on insulin secretion.^41^ Therefore, the relationship of insulin secretion to dopamine needs further inquiry.

The patients with CP groups had lower abundance of sphingolipids compared to controls. These classes of metabolites have been linked to the pathophysiology of metabolic diseases. Studies have shown that insulin resistance in the muscle was characterized by higher levels of sphingolipid molecule C18:0; however, lower concentrations of plasma lysophosphatidylcholine and lysoalkylphosphatidylcholine indicated insulin resistance.^42^ This is an area that needs further evaluation in the context of glycemic aberrations in patients with CP.

Our fecal metabolites analysis revealed less butyrate and propionate in the disease cohort. Butyrate generally helps to suppress intestinal and systemic inflammation. A decrease in its production is linked to worsen gut barrier integrity and increased inflammation, which further can exacerbate pancreatic damage. Both of our microbiota functional analysis and fecal metabolite data revealed reduced concentration of butyrate especially in the patients with diabetes. Another short-chain fatty acids, propionic acid is one of the key products of the microbial fermentation of dietary fiber. Studies have shown that propionate can counteract inflammation of human subcutaneous adipose tissue.^43^ Acetic has been found to be increased in the disease group. Though the role of fecal acetate is still not clear, studies have indicated that decreased levels of serum acetic acid may participate in glucocorticoid induced glycolipid metabolism disorder.^44^ We also observed lower levels of carnitine in the patients with CP. Preclinical animal studies and a few small human trials in related conditions suggested that carnitine may have a protective role in pancreatitis due to its antioxidant and mitochondrial supportive properties. However, clinical data specifically for carnitine treatment in established human CP is limited, and more research is needed.^45^ Overall, the metabolomic alterations in our study appears to align with the jejunal microbial dysbiosis and metabolic derangement (especially diabetes) in patients with CP.

Our study had limitations. The sample size was relatively small and we evaluated data from a single time point. Therefore, the findings may not be generalizable to the entire population of patients with CP. However, this is a pilot study and the data needs to be considered as preliminary which has demonstrated a trend toward contribution of jejunal microbial and plasma/stool metabolomic abnormalities to the development of metabolic derangements in CP. Further validation with larger sample sizes and longitudinal multiple time-point assessments are needed to confirm these findings and elucidate the underlying mechanisms. The strength is that to the best of our knowledge, this is the first study to have evaluated jejunal microbial dysbiosis in patients with CP. We followed strict enrollment criteria and the demographic, nutritional, and disease morphological factors were similar between the patients with and without diabetes. The controls were also cohabiting with the patients for a long duration. Therefore, confounder effect was unlikely to have impacted the dysbiotic and altered metabolomic data in our study. Finally, we evaluated both the microbiota and metabolome (plasma and stool) in our patients. The plasma metabolomic data aligns to our earlier observations,^20^ which validates the current metabolome findings.

In conclusion, our study provides preliminary insights into the jejunal microbial dysbiosis and its potential contribution to the development/progression of metabolic derangements in patients with CP. Further research is needed to validate our findings, explore the mechanistic associations, and identify putative therapeutic targets.

## Supplementary Material

Supplementary Table 1 DNA Quality, Sequence Statistics, Metagenomic Diversity, Bacterial Diversity of the Microbiome Across Groups.

Supplementary Table 2 edgeR_analysis of the Microbiome of the Samples Across the Groups.

Supplementary Table 3 Pathway Profiles Observed (In-Silico) in the Jejunal Aspirate Samples.

Supplementary Table 4 (A) Significantly Altered Plasma Metabolite Profiles of the Groups and (B) Short Chain Fatty Acids (SCFAs) Detected in the Fecal Metabolite Profiles of the Groups.
